# Processability of Thermoelectric Ultrafine Fibers via Electrospinning for Wearable Electronics

**DOI:** 10.1021/acsomega.3c03019

**Published:** 2023-08-09

**Authors:** Elena Ewaldz, Joshua M. Rinehart, Madison Miller, Blair Brettmann

**Affiliations:** † School of Materials Science and Engineering, Georgia Institute of Technology, 711 Ferst Drive, Atlanta, Georgia 30332, United States; ‡ School of Chemical and Biomolecular Engineering, Georgia Institute of Technology, 311 Ferst Drive, Atlanta, Georgia 30332, United States

## Abstract

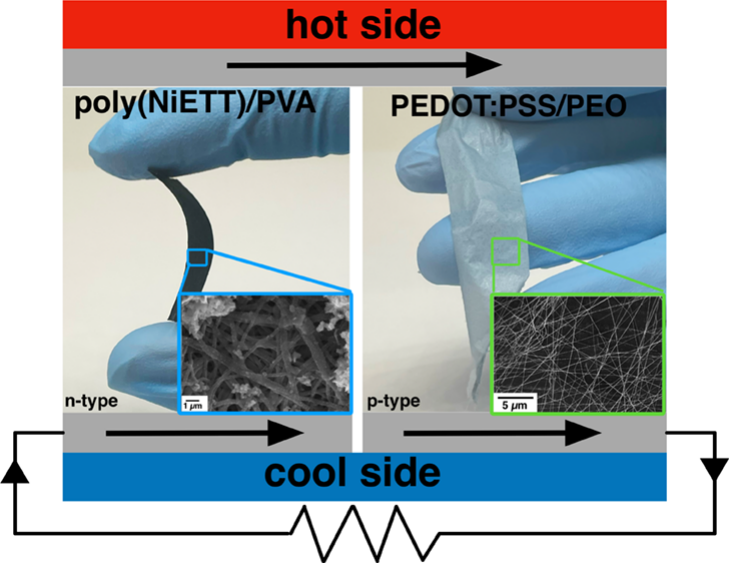

Polymer-based thermoelectric generators hold great appeal in the realm of wearable electronics as they enable the utilization of body heat for power generation. Fibers produced from conducting polymers for use in thermoelectric generators have high porosity and good flexibility, providing comfort-based performance advantages over thin films for wearable electronics. Some fiber processing techniques have been explored to produce textile-based thermoelectric generators; however, they fail to approach the conductivities of polymeric thin films. Ultrafine fibers solution processed through electrospinning yield fiber diameters on the nanoscale, allowing for high surface area to volume ratios and thus low thermal conductivity; however, a number of processing challenges in electrospinning conducting polymers limit the success of preparing high performing thermoelectric textiles. In this work, the specific processing challenges inherent to electrospinning conducting polymers are addressed for both n- and p-type materials. For the p-type polymer, 63 wt % PEDOT:PSS fibers are fabricated through solution formulation improvements yielding a conductivity of 3 S/cm and a power factor of 0.1 μW/mK^2^. The first of their kind n-type poly(NiETT)/PVA electrospun fibers were created yielding a conductivity of 0.11 S/cm and a power factor of 0.0036 μW/mK^2^. These nonwoven ultrafine fiber mats show progress toward achieving textile-based thermoelectric materials with equivalent performance of comparable polymeric thin films. This work shows the feasibility of creating ultrafine fibers for use in thermoelectric generators through electrospinning including the first demonstration of poly(NiETT)/PVA fibers.

## 1 Introduction

Wearable electronics that can be deftly incorporated within or on the human body have attracted interest in recent years. They have been shown to be useful in biomedical,^1,2^ energy harvesting,^3^ and clothing applications.^4^ Yet, a main issue with many of these small wearable electronic components is the requirement of high energy densities with limited options for power sources or battery components. Most commercially available options are either large and bulky or have limited charge storage. As one approach to this problem, there has been increasing interest in thermoelectric (TE) generators. These can convert human body heat into power, enabling constant power generation from the temperature differential between the body and typically cooler air temperatures.^5^

TE generators consist of p- and n-type semiconductors connected electrically in series and thermally in parallel. When a temperature differential is applied, a voltage is produced through the Seebeck effect.^6^ As such, materials with low thermal conductivities and high electrical conductivities are required. TE generators typically consist of inorganic materials including rare earth metal alloys such as bismuth telluride combined with ceramics. These traditional materials are expensive, rigid, and not always biocompatible.^7^ Previous work in conjugated polymers has produced promising organic replacements for traditional TE materials, as they have inherently low thermal conductivity and can be more easily processed at low cost.

Organic textile-based TEs have been of great interest as they have tunable properties, flexibility, and low weight and are comfortable to be worn on the body. Currently, the production of organic TEs is limited due to the strict performance requirements: good mechanical, chemical, and electrical durability; optimal geometric design for greatest power density; and matching n- and p-type conductors.^8^ Therefore, innovative processing methods that enhance these performance requirements should be further developed. Methods developed thus far for creating TE textiles have been deposition on fabrics via screen printing, vapor deposition, and other coating methods.^7^ Though these methods show great promise, they still lack in performance, and applications of textile-based polymer TE materials have been limited. Coatings may abrade and wear over time limiting the long-term efficacy without additional processing steps.^9^ Additionally, coatings require extra steps in production limiting high production capacity. Conductive fibers consisting of poly(3,4-ethylenedioxythiophene)-polystyrene sulfonate (PEDOT:PSS) have been produced via wet-spinning in sulfuric acid that shows electrical conductivities up to 3663 S/cm.^10,11^ We propose an alternative method of creating fibers that are on the nano- to micron-scale that could be used more readily in microfabrications and eliminate the use of corrosive solvents.

Fibers manufactured through electrospinning have high surface areas and high porosity, which can significantly decrease their thermal conductivity. They can also have aligned polymer chains,^12^ near infinite contacts between fibers, and good flexibility and stretchability. Additionally, electrospinning is a continuous processing method allowing for feasible mass production. Electrospinning has shown promise in creating ultrafine fibers from conducting materials such as carbon nanotubes, polyaniline, PEDOT:PSS, and poly(3-hexylthiophene) (P3HT).^13,14^ However, there are limitations in the electrospinning of organic TE materials, as the performance requirements for functional wearable electronics require complex and hard to process solutions. Conducting polymers in the doped form are highly charged, have a relatively low molecular weight for electrospinning or are dispersed as discrete particles, and have high surface tension. These limitations necessitate the addition of a high-molecular-weight polymer that is inherently insulating and thus hinders the electrical conductance of the TE material; therefore, better consideration for fiber production in reducing the inclusion of insulating materials is required to make TE ultrafine fibers more efficient for practical use in wearable devices.

In order to address the development challenges in TE materials, we address the unique processing challenges for both p- and n-type materials. P-type materials such as PEDOT:PSS exhibit solution processing challenges of high surface tension and significant interactions between components in the spinning dope. Recent developments to understand how factors of polymer solutions such as surface tension, solution conductivity, and viscosity impact electrospinning have allowed for production of ultrafine fibers with greater concentrations of functional materials.^15^ In this work, we apply these formulation understandings to study the processability of ultrafine fibers consisting of the p-type material PEDOT:PSS.

N-type materials exhibit their own processing challenge of not being readily solution processable, as many n-type conducting polymers are insoluble in most solvents, have lower electrical conductivities or are not stable in the doped conducting state.^16^ Therefore, little development of solution processing methods has been pursued outside of creating composite films in an insulating polymer matrix.^16,17^ Innovative processing methods of preparing n-type materials are required so that these materials may be developed for wearable electronics. Here, we present a novel method of fabricating an n-type textile using polynickel ethenetetrathiolate (poly(NiETT)) through the synthesis of ultrafine fiber precursor fibers.

The n- and p-type materials, poly(NiETT) and PEDOT:PSS, respectively, were chosen because they can be fabricated via solution processing and have been well studied as high-efficiency organic TE materials.^16,18^ The thermoelectric performance as evaluated by conductivity and Seebeck coefficient shows these to be promising for future use in TE generators to power wearable electronics.

## 2 Experimental Section

### 2.1 Materials

Polyethylene oxide (PEO, 4000 kg/mol), PEDOT:PSS 1.3 wt % dispersion in water conductive grade, ethylene glycol (99%), and Triton X-100 (TX100) were purchased from Sigma-Aldrich and used without further purification for the production of p-type fibers. Nickel(II) acetate tetrahydrate (NiOAc_2_) was purchased from Sigma-Aldrich; polyvinyl alcohol (PVA, 145–180 kg/mol, 88% hydrolyzed) was purchased from Acros Organics for the electrospinning of n-type precursor fibers. Methanol was purchased from Fisher Scientific and thoroughly degassed with argon and dried over 3 Å molecular sieves before use. 1,3,4,6-Tetrathiapentalene-2,5-dione was purchased from TCI America and recrystallized from acetonitrile before use, yielding light tan needle-like crystals. Sodium hydroxide and glacial acetic acid were purchased from VWR BDH and used as received. Crystalline iodine was used as received from Alfa Aesar.

### 2.2 Preparation of p-Type Spinning Solutions

The preparation of p-type polymer solutions for use in electrospinning is as follows. 75 mg of PEO, 500 mg of ethylene glycol, and 10 mg of TX100 were combined in 10 g of PEDOT:PSS aqueous dispersion. The solution was mixed with magnetic stirring in a 10 °C water bath for at least 48 h for complete dissolution. The solution was then sonicated using a sonication probe with an attached microtip for 10 s.

### 2.3 Preparation of p-Type Fibers

The electrospinning setup contained a parallel plate geometry, a syringe pump, a ES30P-5W Gamma High Voltage power supply, and a personal home use hairdryer. Fibers were electrospun from solution at room temperature and unregulated humidity ranging between 48 and 56% RH. The flow rate was 0.6 mL/h, voltage was 10 kV, and the plate distance was 25 cm. The hairdryer was positioned above the top plate with downward flow (low heating setting) to prevent freestanding fibers from accumulating due to the high conductivity. Samples were spun directly onto silicon wafers with a 200 nm oxide layer and clean glass slides for later characterization.

### 2.4 Preparation of Ni/PVA Acetate Precursor Fibers

To prepare precursor fibers for the n-type material, 6 wt % NiOAc_2_ was mixed with 6 wt % PVA in deionized water. Solutions were magnetically stirred at room temperature for 48 h for complete dissolution. Fibers were spun with the same setup as the p-type fibers but with no forced air. The flow rate was 0.3 mL/h, voltage was 21 kV, and the plate distance was 25 cm. The resulting fiber mat was kept in a desiccator until the polymerization reaction.

### 2.5 Polymerization of Poly(Na(NiETT)) Fibers

The reaction to create poly(Na(NiETT)) fibers is as follows. All glassware was dried in an oven before use. The PVA/NiOAc_2_ mat was dried under vacuum overnight at 60 °C. All procedures are done using Schlenk line and air-free procedures. 156 mg of the PVA/NiOAc_2_ mat was folded and placed in a round-bottom flask and thoroughly purged with argon. 156 mg of PVA/NiOAc_2_ mat contains 52 mg of NiOAc_2_·4H_2_O (2:1 PVA:NiOAc_2_·4H_2_O by weight). Equates to 2.09 × 10^–4^ moles of Ni^2+^ ions. 0.044 g of thiapendione (2.11 × 10^–4^ moles, 1 equiv) was added to a pear-shaped flask with 1.5 mL of dry MeOH and stirred at 60 °C. 0.042 g of NaOH (1.06 × 10^–3^ moles, 5 equiv) was added to another pear-shaped flask with 1.5 mL of dry MeOH. After dissolution of NaOH, NaOH solution was added to the thiapendione via cannula and stirred at 60 °C for 24 h. After 24 h, 2 mL of MeOH was added to PVA/NiOAc_2_ via a syringe. The thiapendione solution was added to PVA/NiOAc_2_ round bottom via cannula. The previously green fiber mat changed color to black. The fiber mat was covered with solution and gently stirred for 24 h. 0.054 g I_2_ (2.11 × 10^–4^ moles, 2 equiv) was dissolved in 1.25 mL MeOH in a pear-shaped flask. 0.088 mL of glacial acetic acid was pipetted into a pear-shaped flask and 1.25 mL of MeOH added, followed by degassing the solution with argon. Glacial acetic acid was added to the PVA/NiOAc_2_ mat via cannula and shaken lightly to ensure all of mat is covered. Immediately after, iodine solution was added to PVA/NiOAc_2_ mat via cannula and allowed to stir at 60 °C for about 6 h. The fiber mat was washed with MeOH by adding about 30 mL of MeOH, stirring and allowing to sit for 30 min, then decanting, and adding fresh MeOH three times. The fourth wash was performed with ethyl ether, and a final fifth wash was with deionized water. The round-bottom flask was put on a roto-evaporator to dry the material and any remaining solvent.

### 2.6 Solution Characterization

Solutions were characterized for parameters related to their electrospinnability. Solution conductivities were obtained using a VWR symphony meter. Zero shear viscosity measurements were performed on a TA Instruments DHR-3 rheometer using a double-gap cylinder geometry. The measurements were taken at 25 °C using a shear rate sweep between 1 and 1000 s^–1^. Surface tension measurements were obtained using the pendant drop method with a DataPhysics goniometer using 10 different drops. Extensional viscosity measurements were performed using the dripping-on-substrate method on an in-house setup. The diameter of the nozzle used was 1.55 mm, and the flow rate was set to 1 mL/h. Videos of the thinning capillary bridge were captured at a frame rate of 8000 fps using a high-speed camera (Chronos 1.4) with a 12.5–75 mm f/1.2 zoom lens and super macro lens. The videos were analyzed using ImageJ and MATLAB.

### 2.7 Morphology Characterization

Scanning electron microscopy (SEM) micrographs were acquired on samples using a Zeiss Ultra60 FE-SEM. Samples were coated with gold using a Hummer 6 sputterer at 15 mA for 1 min prior to imaging. Cross-sectional samples were prepared on a 90° angle stub. ImageJ was used to measure the thickness of cross-sectional samples.

### 2.8 X-ray Photoelectron Spectroscopy (XPS)

XPS was performed on poly(NiETT)/PVA fiber mats using a Thermo K-Alpha XPS (Thermo Fisher Scientific Inc.).

### 2.9 Thermoelectric Characterization

Prior to testing thermoelectric properties, samples were affixed to glass slides, and 200 nm thick gold contact pads were deposited using a CHA E-beam evaporator in a 4 mm × 4 mm square configuration. Samples were placed on a temperature-controlled Peltier stage, and conductivities were obtained using the van der Pauw measurement method performed with a Keithley 2700 DMM with a 7708 Mux card via a LabVIEW interface. Conductivities were measured at 2.5 °C intervals between 20 and 35 °C. The Seebeck coefficient (*S*) was measured by placing the sample between two temperature-controlled Peltier units and applying a series of temperature differences centered at 25 °C between the stages and measuring the thermoelectric voltages between the stages. The Seebeck coefficient was obtained as the slope of the *V*–Δ*T* plot.

## 3 Results and Discussion

### 3.1 Electrospinning of the p-Type Material

**Figure 1:**
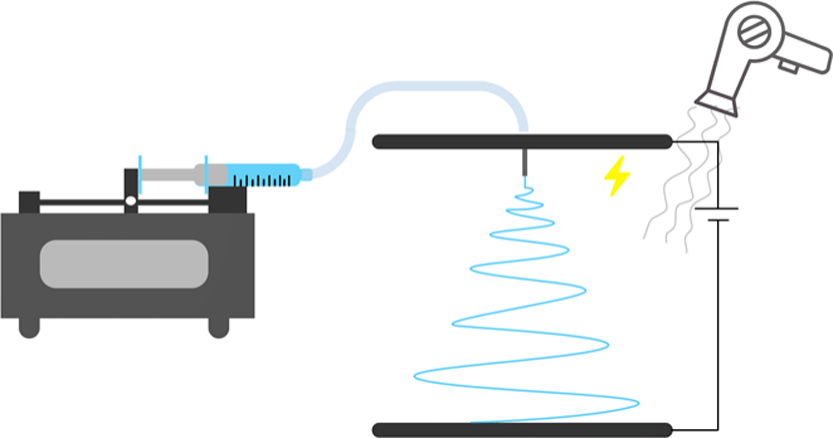
Schematic of electrospinning setup used for p-type fiber fabrication.

**Figure 2:**
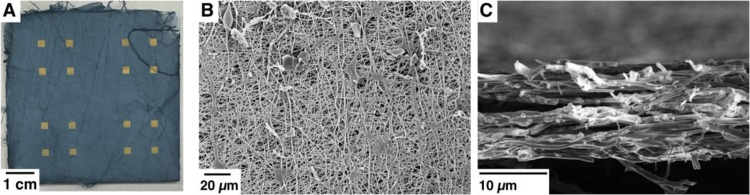
(A) Electrospun PEDOT:PSS/PEO mat on the glass slide for thermoelectric measurements, (B) SEM micrograph of PEDOT:PSS/PEO fibers showing smooth fiber morphology and high porosity, and (C) SEM micrograph of the cross-sectional sample used in fiber mat thickness measurements.

Electrospinning was performed on a custom-built setup with a high-voltage supply, a syringe pump, conducting parallel plates, and a low heated air supply (Figure 1). The air supply was required as highly conducting materials stand up on the grounded collector changing the geometry of the electric field and yielding nonuniform self-assembled shapes.^19^ A standard stationary collector plate was chosen as it yields an unaligned, nonwoven mat, which inherently has very high porosity showing the lower bound for TE performance. Fibers were allowed to collect until a thickness was achieved where the substrate was completely covered (Figure 2A).

Electrospinning typically requires high entanglements to maintain jet cohesion during the fiber formation process;^20^ therefore, PEDOT:PSS cannot form fibers alone as it is a low-molecular-weight suspension and the inclusion of a high-molecular-weight polymer is necessary. In order to maximize the PEDOT:PSS content in the resulting fibers, a high-molecular-weight (4 × 10^6^ g/mol) PEO was mixed with the aqueous PEDOT:PSS dispersion. This molecular weight was chosen because the higher the carrier polymer molecular weight, the lower the concentration required to form uniform fibers.^20^ Due to the high surface tension and low volatility of water, it can be difficult to electrospin these PEO solutions, especially when combined with complex polymer solutions. Therefore, tuning of the polymer solution properties was required in order to enhance the performance.

We have previously studied the impact of surface tension on the electrospinnability of polymer solutions.^15^ Results showed that when surface tension is reduced, lower concentrations of a high-molecular-weight polymer are required to form smooth fibers. Here, TX100, a nonionic surfactant, was used to lower the surface tension of the PEDOT:PSS/PEO mixtures from 58.6 to 31.3 mN/m (Table 1 includes all measured solution properties, including surface tension). Decreasing the surface tension allowed for smooth fiber formation (seen in Figure 2B) of PEDOT:PSS/PEO fibers at a PEO concentration of 0.75 wt % resulting in a concentration of 63 wt % PEDOT:PSS in the final solid fibers. Additionally, ethylene glycol (EG) was included at 5 wt % as it has been shown to increase the conductivity of solution-processed PEDOT:PSS films and does not hinder the ability to electrospin fibers.^21^

**Table 1:** Measured Zero Shear Viscosity (η_0_), Terminal Steady-State Extensional Viscosity (η_E∞_), Surface Tension (γ), and Solution Conductivity (σ) of 0.75 wt % PEO, 0.75 wt % PEO with 0.1 wt % TX and 5 wt % EG, 1.3 wt % PEDOT:PSS with 0.75 wt % PEO, and 1.3 wt % PEDOT:PSS with 0.75 wt % PEO, 0.1 wt % TX100, and 5 wt % EG in DI Water

	η_0_ (Pa s)	η_E∞_ (Pa s)	γ (mN/m)	σ (μS/cm)
PEO	0.14 ± 0.02	270 ± 35	61.8 ± 0.3	68.4
PEO with TX100, EG	0.16 ± 0.02	120 ± 22	31.3 ± 0.1	133
PEDOT:PSS with PEO	0.34 ± 0.10	360 ± 61	58.6 ± 0.4	7420
PEDOT:PSS with PEO, TX100, EG	0.41 ± 0.10	310 ± 25	31.3 ± 0.3	7490

As fiber formation is mostly driven by the rheology of the polymer solutions, the addition of PEDOT:PSS, TX100, and EG was evaluated for their impact on both the shear and extensional rheology. Each additive was evaluated for the impact on the zero shear viscosity (η_0_) (Table 1). PEDOT:PSS doubled the η_0_ from 0.14 Pa s for the pure PEO solution to 0.34 Pa s for the PEO and PEDOT:PSS mixture. This increase is likely caused by the addition of solute and greater interactions between PEO and PEDOT:PSS chains. TX100 and EG slightly increase the shear viscosity, due to their inherent higher viscosities, yet it is not a significant impact. This increase is still within the normal range of electrospinnable solutions^22^ and will have little impact on this solution processing method.

**Figure 3:**
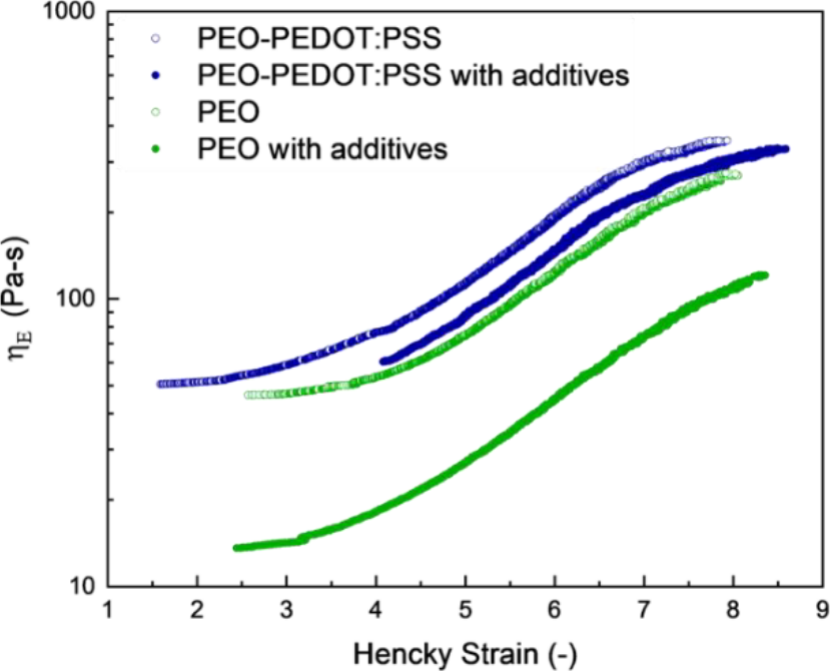
Extensional viscosity vs Hencky strain of 0.75 wt % PEO (open green symbols), 0.75 wt % PEO with 0.1 wt % TX and 5 wt % EG (closed green symbols), 1.3 wt % PEDOT:PSS with 0.75 wt % PEO (open blue symbols), and 1.3 wt % PEDOT:PSS with 0.75 wt % PEO, 0.1 wt % TX100, and 5 wt % EG (closed blue symbols) in DI water.

Extensional rheology must also be evaluated, as fiber formation occurs during extensional flow with strong elastic forces required to maintain jet cohesion. To study the impact of solution components on the extensional rheology, we utilized dripping-onto-substrate (DoS) rheometry.^23^ DoS allows for the analysis of self-thinning extensional flow pinch-off dynamics, representative of what happens in an electrospinning jet. Through this, we can study how PEO, PEDOT:PSS, EG, and TX100 affect the elasticity of the solution, which is what stabilizes the jet against capillary breakup. Evaluating the extensional viscosity vs Hencky strain, the terminal steady state extensional viscosity (η_E∞_) can be obtained from the plateau reached at high strain values. This serves as the best metric to directly compare to electrospinnability since high electric stress creates very high strain during fiber formation. The extensional viscosity decreases with the addition of TX100 as extensional viscosity is directly dependent on surface tension η_E_ = γ/(ε̇*R*) where γ is the surface tension, ε̇ is the extension rate, and *R* is the radius of the thinning filament/jet. This is directly seen in Figure 3 where the addition of EG and TX100 to PEO decreases the η_E∞_ by roughly the same decrease as the decrease in surface tension (ratio of surface tension without/with additives is 2.0, while the ratio of η_E∞_ without/with is 2.25). For the solutions with PEDOT:PSS, the decrease in η_E∞_ with addition of EG and TX100 is much less significant (ratio of surface tension values is 1.9 without/with additives, while the ratio of η_E∞_ without/with additives is only 1.2). This is likely because the addition of PEDOT:PSS to PEO induces higher intermolecular interactions and includes a greater amount of solute in the system. This leads to an overall higher η_E∞_ and less sensitivity to the surface tension. These interactions alter the intrinsic polymer dynamics, which decreases the extension rate in this self-thinning system. Therefore, future fine tuning of the formulation could potentially allow for even less PEO to be included while still forming smooth fibers by finding additives that decrease the interaction between PEO and PEDOT:PSS.

Morphologies were observed using SEM to confirm fiber formation, evaluate uniformity, and measure the mat thickness. Multiple samples (SI Figure S2) from different mats were taken to examine fiber morphology and uniformity. Fibers were found to be smooth and with a fairly broad distribution of fiber diameters of 333 ± 114 nm. The broad distribution can be attributed to the high conductivity causing fiber clumping before complete drying and some gelation seen in solution (SI Figure S3).

Additionally, measurements of the thickness of fiber mats are required for accurate conductivity measurements. Measuring the thickness of ultrafine fiber mats is difficult due to the fibrous and porous nature of these mats. Most measurement methods for thin films such as profilometry drag fibers causing bunching or depression of fibers, leading to inaccurately high or low measurements. We found that the most effective method is through cross-section examination with SEM (Figure 2C and SI Figures S4, S5). Measurements across five samples yielded a thickness of 14 ± 2.3 μm.

**Figure 4:**
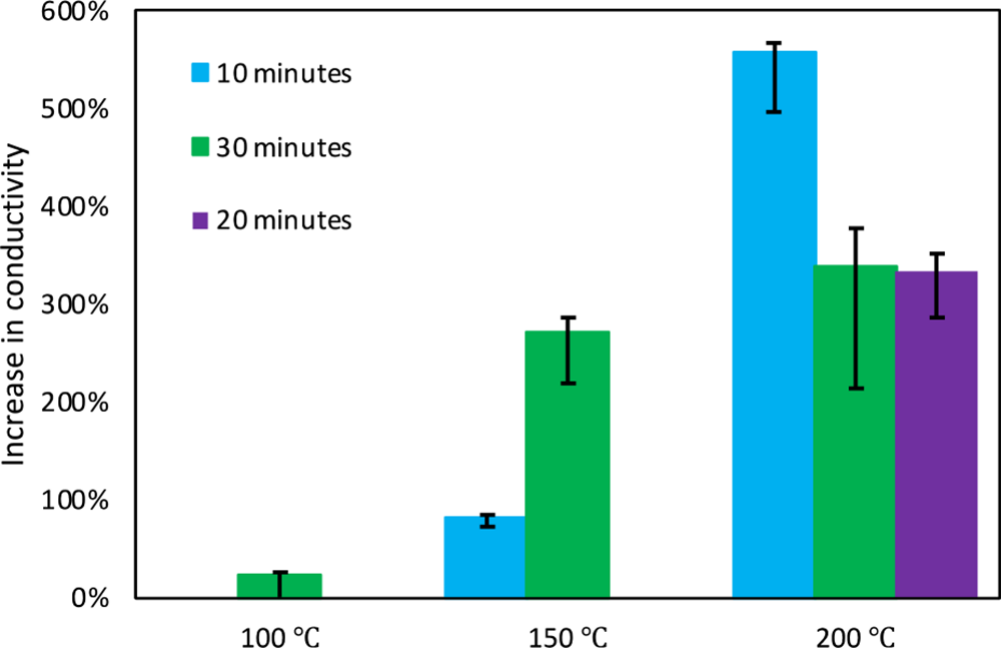
Increase in PEDOT:PSS/PEO fiber mat conductivity with thermal treatment at 100, 150, and 200 °C.

Organic TE materials commonly undergo postprocessing steps to increase the conductivity, such as solvent treatment or thermal treatment. One step of postprocessing was performed to both increase electrical conductivity (σ) and increase the stability of the p-type fibers. Concern over changing the fiber morphology prevented the use of well-studied sulfuric acid post-treatment due to the delicate nature of ultrafine fibers.^10,24,25^ Thermal annealing the fibers simultaneously improves the thermoelectric properties and stabilizes the fibers against water degradation. It is thought that annealing the fibers produces a condensation reaction between PSS and PEO at high temperatures, creating water-resistant crosslinks and allowing for better charge transport along the PEDOT chain.^26,27^ Thermal annealing has been found to be optimal for PEDOT:PSS between the ranges of 100 and 250 °C with chemical degradation occurring at higher temperatures.^28−30^ We evaluated three different annealing temperatures and two times, with an additional time of 20 min at 200 °C, Figure 4. We determined that the greatest enhancement was seen at 200 °C for 10 min (Figure 4). Additional time above 10 min resulted in a less of an increase in conductivity (green and purple bars in Figure 4), which could be a result of mild degradation beginning to occur. Values for conductivity and Seebeck coefficient reported in this work follow this 10 min, 200 °C thermal annealing processing step. Additionally, we observed a significant increase in fiber stability when the annealed fibers were immersed in water.

### 3.2 Electrospinning of the n-Type Material

**Figure 5:**
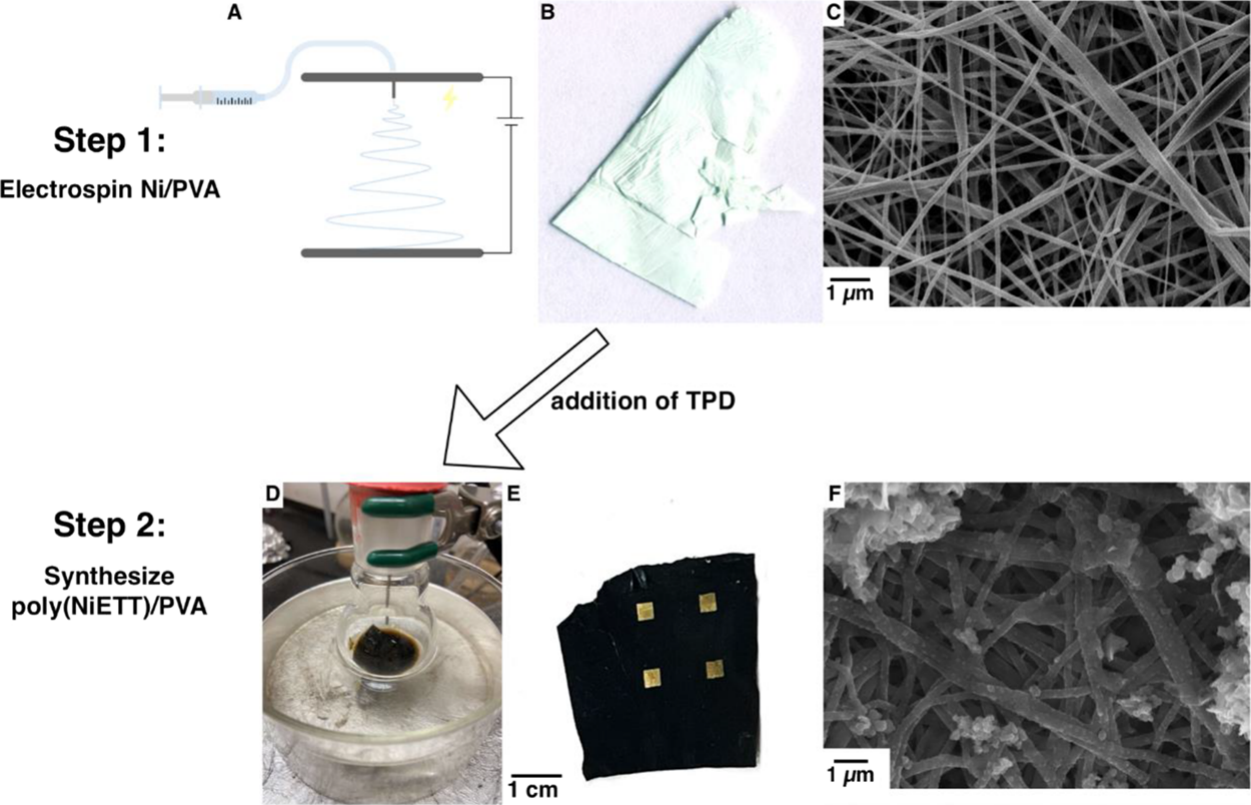
Production of n-type thermoelectric material poly(NiETT)/PVA. (A) Electrospinning setup, (B) nickel acetate/PVA precursor fibers showing light green color, (C) SEM micrograph of nickel acetate/PVA precursor fibers, (D) fiber mat in flask after addition of thiapendione showing color change, (E) poly(NiETT)/PVA fiber mat after drying with gold contacts, and (F) SEM micrograph of poly(NiETT)/PVA synthesized.

In addition to preparation of p-type fibers, we aim to prepare n-type fibers for TE textiles. Pure NiETT particles are not readily electrospinnable, nor do they provide optimum electron transport in a fiber; therefore, for the n-type material, a novel procedure for poly(NiETT) synthesis was developed through the use of PVA and nickel salt precursor fibers, illustrated in Figure 5. NiETT/PVA has been shown to form a fully complexed composite structure within a surface layer when nickel acetate is solubilized in PVA and reacted with tetrathiooxalate.^31^ PVA is readily electrospun from water and forms smooth uniform fibers at 6 wt % in DI water.^15^ Nickel salts can be electrospun in PVA in large amounts, though at higher amounts excess nickel salt crystallizes on the outside of fibers and will react to form NiETT in solution rather than it forming within the fibers. Nickel acetate was fully dissolved in a solution of PVA in DI water in a ratio of 1:1 nickel acetate:PVA. The Ni/PVA precursor fibers were reacted fully and dried while pressing flat.

The full synthesis of poly(NiETT)/PVA was evaluated through the distinct color change in the fiber mat, along with XPS analysis. As-spun nickel salt PVA fibers appear as a pale green fiber mat (Figure 5B). During the synthesis of poly(NiETT)/PVA the fiber mat changes to black, indicative of poly(NiETT) formation. Further characterization of poly(NiETT)/PVA was performed using XPS (SI Figure S1). Distinct peaks are observed at ∼854 eV corresponding to the primary Ni-S binding environment and ∼856 eV corresponding to Ni coordinating with oxygen either between PVA or with byproducts from the reaction.^16^ Additionally a small peak is seen at ∼860 eV, which verifies that the nickel is in a square-planar coordination.^32^

The morphology of n-type fibers was observed for both the precursor fibers and after synthesis. At all nickel acetate loadings in PVA, fibers were observed to be smooth and uniform with uniform fiber diameters of 140 ± 27 nm (Figure 5C). No separation between the nickel and PVA was observed, indicative of a fully solubilized and uniformly mixed system, as has been shown to occur with electrospun fibers with small molecules.^33^ After synthesis of poly(NiETT)/PVA, fiber diameters increase slightly to 215 ± 37 nm and present with significant roughness as seen in Figure 5F. This roughness was observed previously in thin films and attributed to potential Ni^2+^ migration to the surface of thin films leading to a higher concentration of poly(NiETT)/PVA on the surface.^31^ As the fibers are on the nanoscale, it is reasonable to assume that the same phenomenon is occurring.

### 3.3 Thermoelectric Properties

Once both p-type and n-type materials were prepared, samples were affixed to clean glass slides and three separate locations had gold contacts deposited to ensure good electrical contact and mitigate surface roughness and contact resistance at the probe locations. A four-point probe setup was used to measure the bulk TE properties, which are listed in Table 2. Our fibers showed a conductivity of 3.00 +/– 0.06 S/cm, while previously electrospun PEDOT:PSS with PEO or other carrier polymer fibers have conductivities ranging on the orders of 10^–8^ to 10^–2^ S/cm.^13^ The high concentration of PEDOT:PSS in our final bulk fiber mat leads to this substantial increase in conductivity, as high as 3.0 S/cm along with the improvements from thermal annealing that were discussed previously. As expected, the inherent high porosity of ultrafine fiber mats and addition of PEO to allow for electrospinning yielded TE properties that were lower than what is typically found for pure PEDOT:PSS thin films, with conductivities of 70 and 300 S/cm for undoped and doped, respectively.^34,35^

**Table 2:** Thermoelectric Properties of the p- and n-Type Polymer Thermoelectric Materials as Thin Fibrous Mats on Glass at Room Temperature

	*S* (μV K^–1^)	σ (S cm^–1^)	*S*^2^σ (μW m^–1^ K^–2^)
p-type	18.5 ± 0.4	3.00 ± 0.06	0.100 ± 0.003
PEDOT:PSS/PEO			
n-type	–18.4 ± 3.4	0.11 ± 0.03	0.0036 ± 0.0007
poly(NiETT)/PVA			

The Seebeck coefficient of our ultrafine fiber mats of 18.5 μV/K is slightly lower than that found for thin films of 20 μV/K.^34^ The intrinsic pores in the fiber mats act as impurities inhibiting the charge transport through the bulk of the material resulting in a decreased Seebeck coefficient. While achieving a high Seebeck coefficient is necessary to achieve a high power factor, the high porosity can significantly decrease the thermal conductivity. The porosity influence on thermal conductivity could lead to a greater TE figure of merit (*ZT*) compared to that of equivalently composed thin films as *ZT* = (*S*^2^σ)/*kT*, where *k* is the thermal conductivity and *T* is the absolute temperature. The combined conductivity and Seebeck coefficient lead to a power factor of 0.1 μW m^–1^ K^–2^, which substantially outperforms previous PEDOT:PSS fabrics such as a PEDOT:PSS-coated polyester with a power factor of 0.045 μW m^–1^ K^–2^.^36^

Further enhancement of PEDOT:PSS ultrafine fibers can be achieved through other doping or post-treatment steps or a further decrease in the amount of the insulating PEO. These enhancements could make the fibers rival the TE performance of thin films while still maintaining the benefits of low thermal conductivity and high breathability of ultrafine fiber mats.

**Figure 6:**
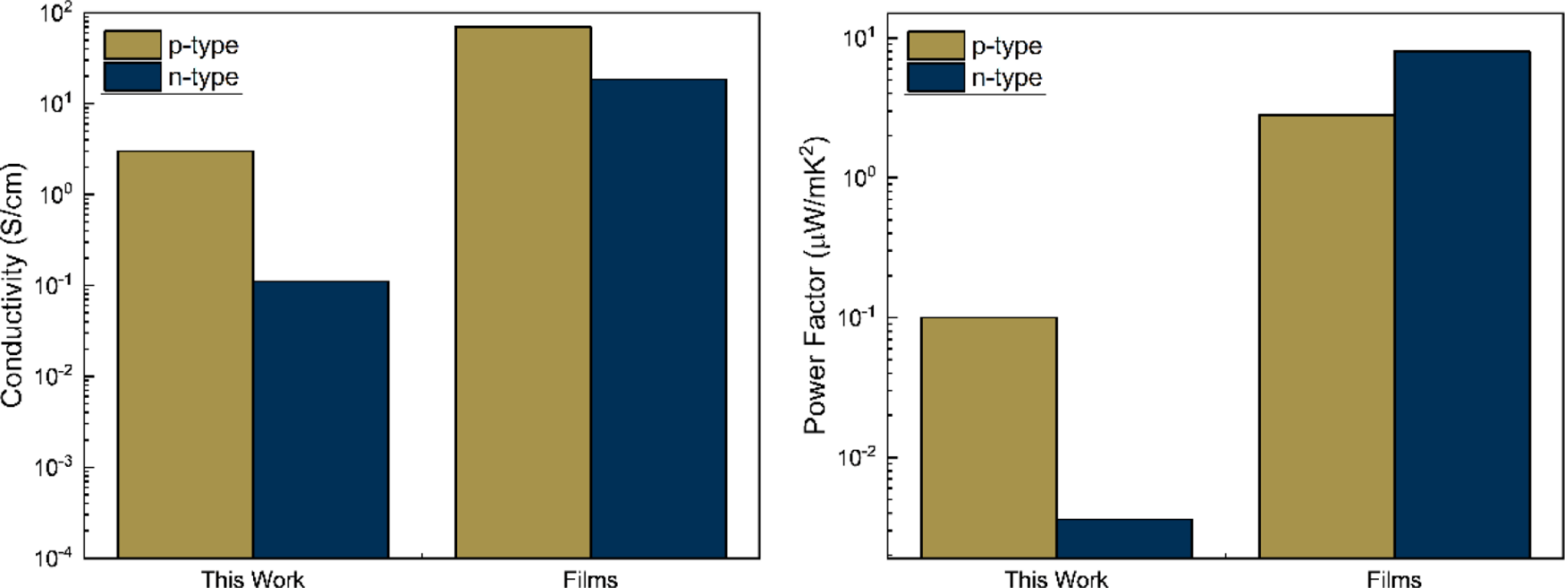
Comparison of conductivity and power factor of the ultrafine fiber mats in this work to polymeric thin films of pure PEDOT:PSS and poly(NiETT)/PVDF films measured using the van der Pauw measurement method. Data plotted for films are from ref (34).

Due to the limited work in n-type polymeric materials, to our knowledge there is only one other work that has created organic n-type polymer nanofibers by electrospinning.^37^ Poly(*N*,*N*′-bis(2-octyl-dodecyl)-1,4,5,8-napthalenedicarboximide-2,6-diyl-alt-5,5′-(2,2′-bithiophene)) (N2200), a commercially available n-type polymer with a rigid π-conjugated backbone, was electrospun with PEO as a sacrificial carrier polymer. The chemical structures of N2200 and poly(NiETT) are shown in Supplementary Information Figure S6. This N2200 nanofiber mat yielded a maximal conductivity of 7.06 × 10^–4^ S/cm, a Seebeck coefficient of −346 μV/K, and a power factor of 0.0085 μW/(mK^2^) and was able to reach the performance of thin films with the addition of a dopant. Our poly(NiETT)/PVA fibers show a significant improvement of conductivity with an average of 0.11 S/cm and again show advancement to reaching the conductivity of 18.4 S/cm for comparable poly(NiETT) thin films.^34^ The Seebeck coefficient of our poly(NiETT)/PVA n-type fibers was −18.4 μV/K compared to −66 μV/K for poly(NiETT)/PVDF thin films.^34^ The combined conductivity and Seebeck coefficient lead to a power factor of 0.0036 μW/(mK^2^) comparable to the N2200 nanofiber mat. In the future, TE property enhancement in poly(NiETT)/PVA fibers can be achieved through further evaluation of poly(NiETT) concentrations and inclusion of dopants. Postprocessing steps could also be pursued as thermal annealing has been shown to enhance performance for NiETT/PVDF thin films though the mechanism for this enhancement may be different than what is possible with our composite fibers.^17^ The comparison of the electrospun mats in this study to typical polymeric thin films is summarized in Figure 6.

**Figure 7:**
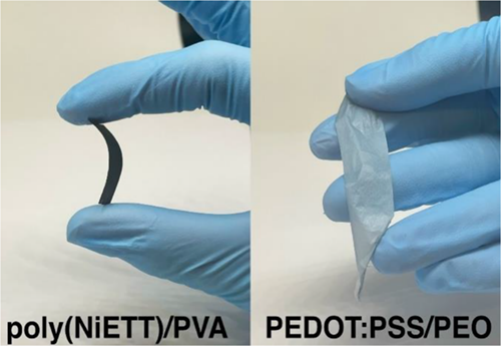
Images of free-standing thermoelectric ultrafine fiber mats for n-type (poly(NiETT)/PVA) and p-type (PEDOT:PSS/PEO) materials.

These electrospun ultrafine fiber mats are compatible with textile-based thermoelectrics and wearable electronic devices as they are flexible, self-standing (Figure 7), stretchable, breathable, and readily shaped to various structures. These factors allow for comfortable next-to-body applications and adaptability with human body movement.

## 4 Conclusions

Electrospinning is a promising method of preparing TE ultrafine fiber mats as they have a high surface area allowing for increased charge transport and are highly porous which is desired for near or on the body applications. However, developing these organic TE yields numerous processing challenges specific to p-and n-type materials. P-type polymers are solution-processable but require the addition of an inert polymer to form fibers. Limiting the inert polymer is desired for high performance but creates a high surface tension and highly interacting solution making electrospinning impractical. We addressed these issues in electrospinning PEDOT:PSS with the addition of a high-molecular-weight PEO, a surfactant TX100, and a conductivity enhancing additive, EG, resulting in a PEDOT:PSS concentration of 63 wt %, which to our knowledge is the highest reported in ultrafine fibers. Thermal treatment was employed to enhance TE properties and resulted in a power factor of 0.1 μW m^–1^ K^–2^, exhibiting improved performance over previously reported fabrics.

N-type materials have vastly lacked in development due to difficulties in processing outside of composite films. A novel method was presented for creating n-type ultrafine fibers through the synthesis of poly(NiETT)/PVA complexed fibers. These fiber mats resulted in a power factor of 0.0036 μW m^–1^ K^–2^, which is comparable to the only other n-type ultrafine fiber mat reported.^37^ This method of creating an n-type textile could allow for better development in the field of thermoelectric textiles.

These free-standing, flexible porous mats that demonstrate good TE performance show that electrospinning TE materials can drive the development of textile-based wearable electronics.

## Supporting Information Available

XPS plot, fiber morphology micrographs, and fiber mat cross-section micrographs (PDF)
