# Supplementary material for: Processability of Thermoelectric Ultrafine Fibers via Electrospinning for Wearable Electronics
Source: ACS Omega. 2023 Aug 9;8(33):30239–46. doi: 10.1021/acsomega.3c03019 (PMC10448483; doi:10.1021/acsomega.3c03019)
Supplement: Supplementary file 1 — ao3c03019_si_001.pdf [file ao3c03019_si_001.pdf]

Supplemental Information

Processability of thermoelectric ultrafine fibers via electrospinning for wearable electronics

Elena Ewaldz,[a] Joshua M. Rinehart,[a] Madison Miller,[a] and Blair Kathyrn Brettmann[a,b]*

[a.] School of Materials Science and Engineering, Georgia Institute of Technology, 711 Ferst Drive, Atlanta, Georgia 30332, United States

[b.] School of Chemical and Biomolecular Engineering, Georgia Institute of Technology, 311 Ferst Drive, Atlanta, Georgia 30332, United States

1. XPS analysis of poly(NiETT)

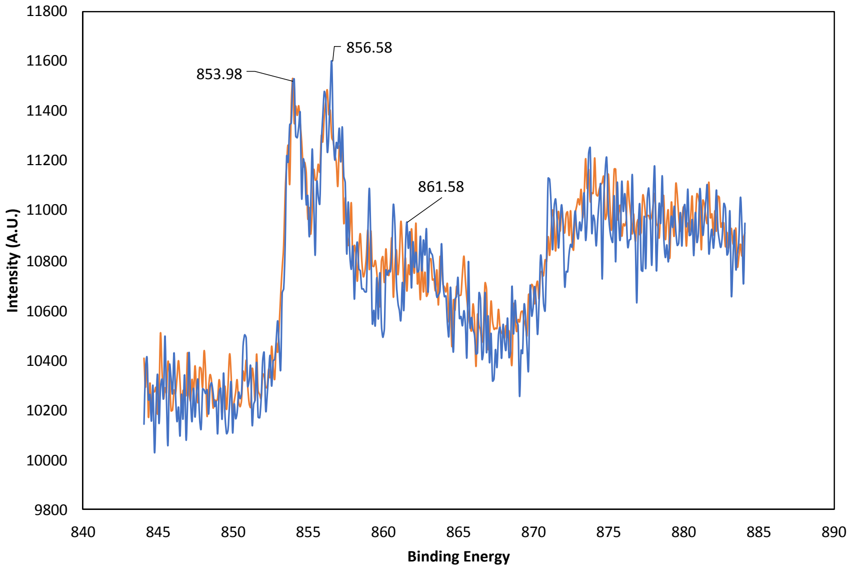

Figure S1. XPS analysis of poly(NiETT) fibers noting peaks at 854, 856, and 861 kV.

2. Fiber morphologies

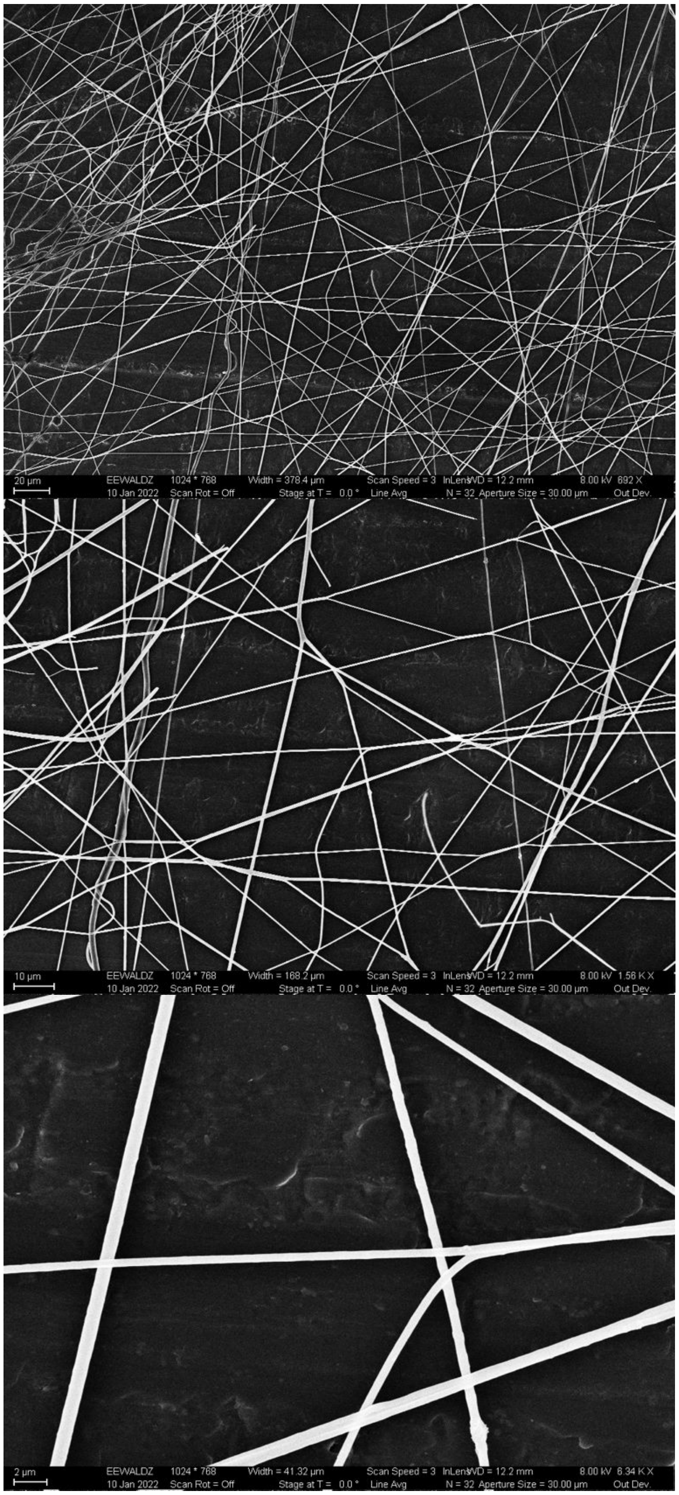

Figure S2. PEO/PEDOT:PSS fiber morphology images as obtained through SEM as used for fiber diameter measurements.

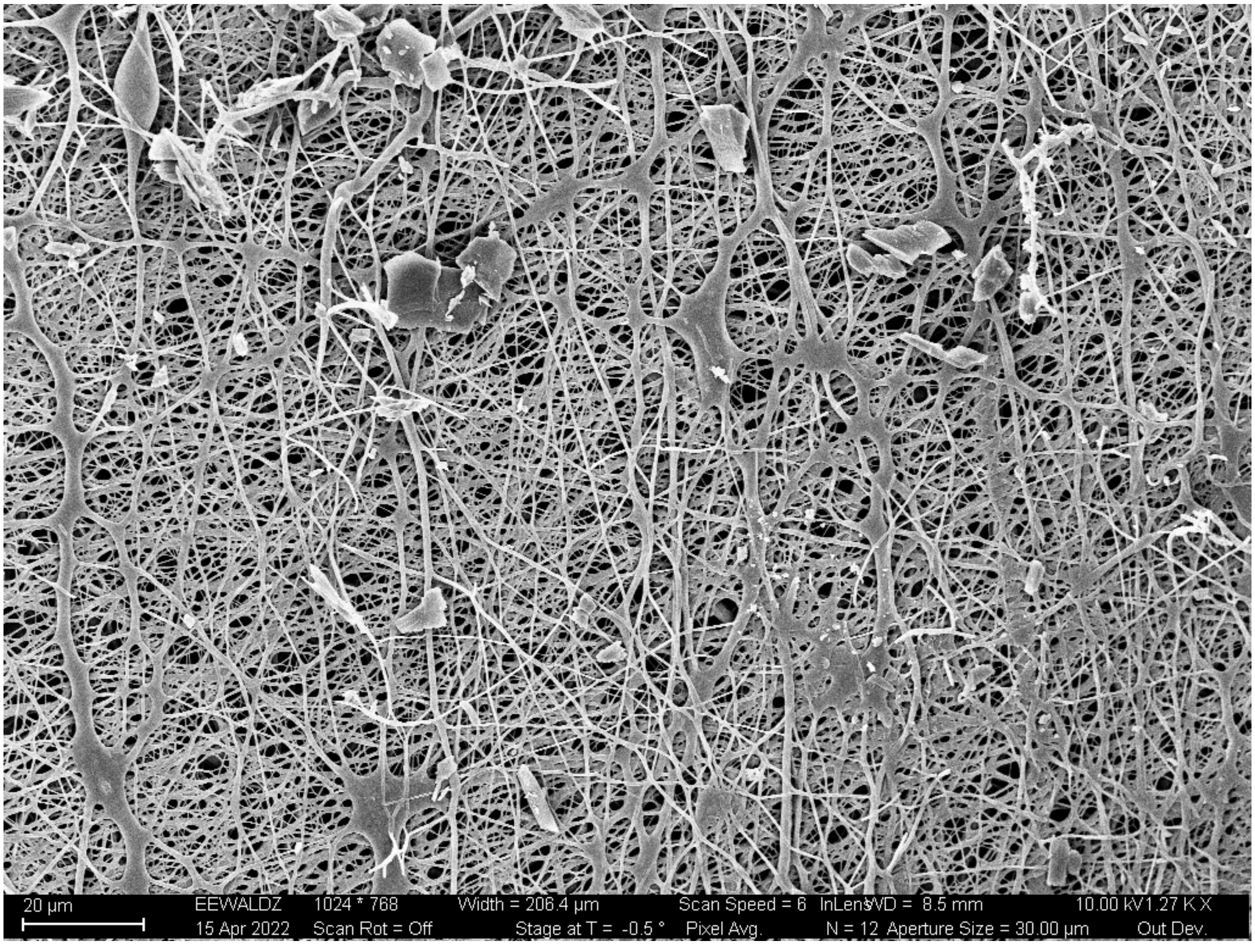

Figure S3. Fiber morphology of PEO/PEDOT:PSS mat for thick sample showing fiber clumping.

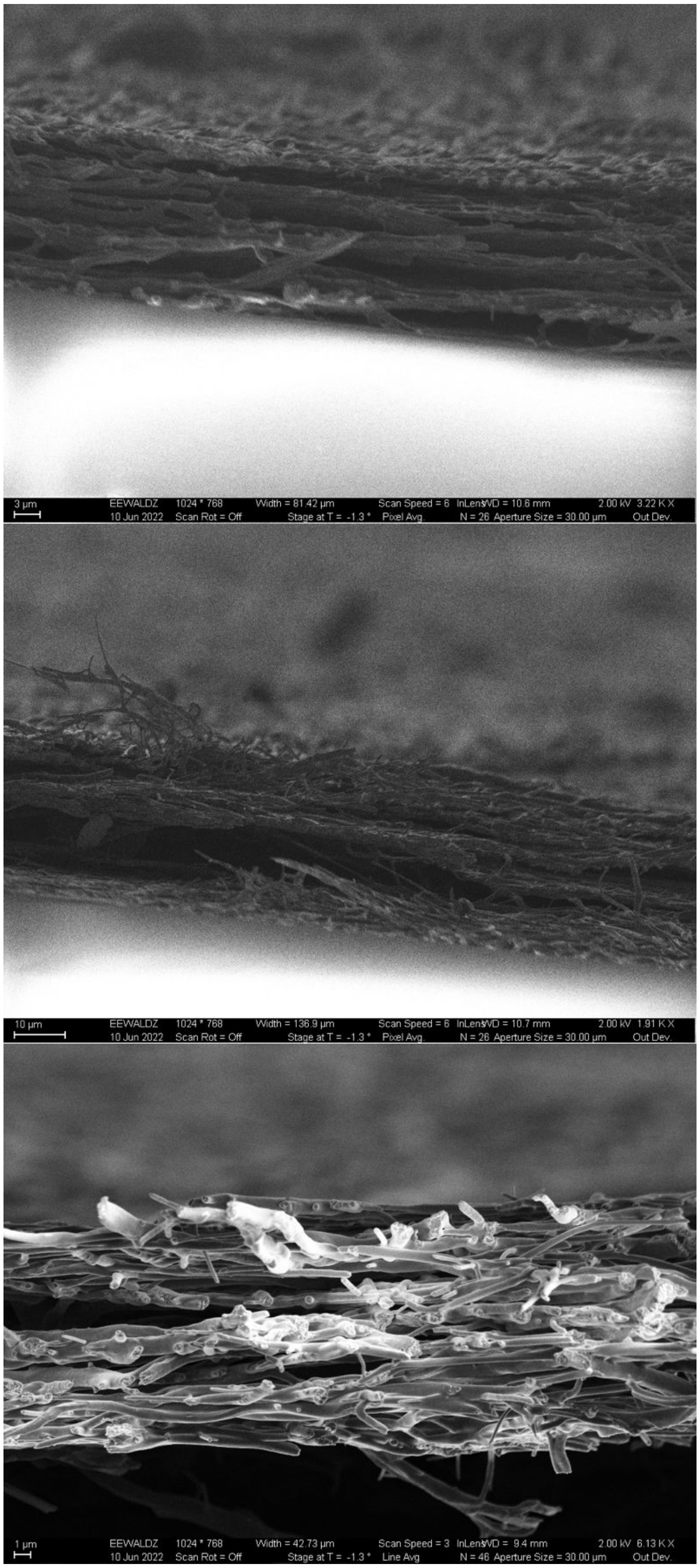

Figure S4. Cross sectional images of PEO/PEDOT:PSS fiber mats used for thickness measurements

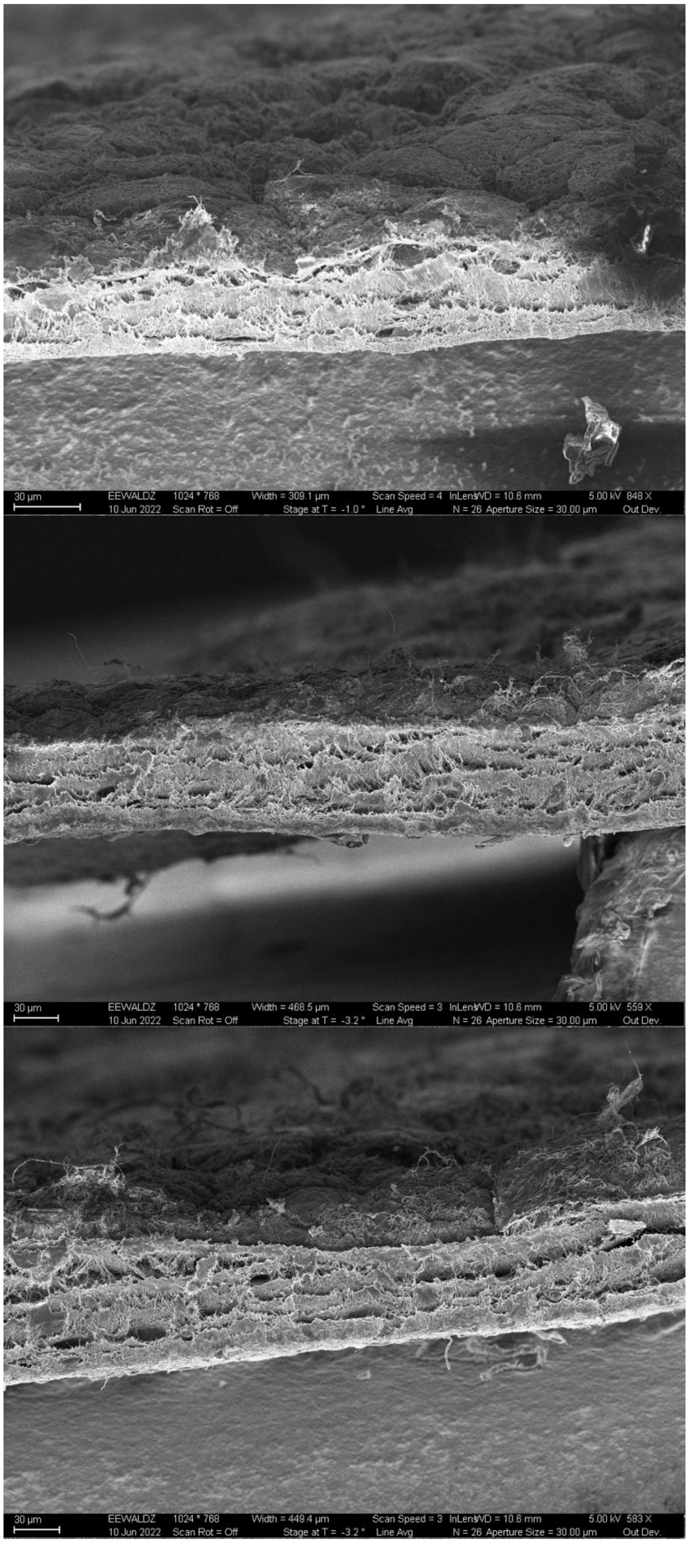

Figure S5. Cross sectional images of PVA/poly(NiETT) fiber mats used for thickness measurements

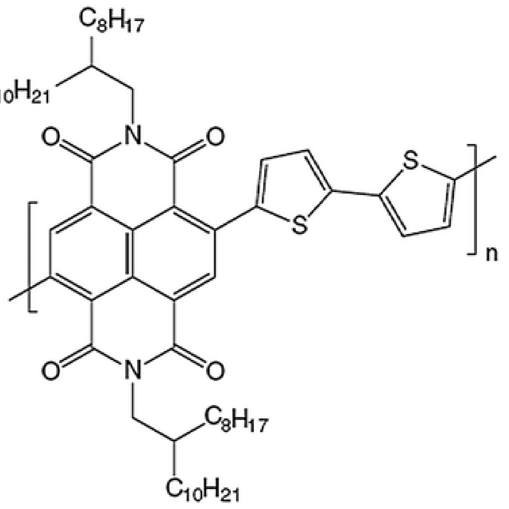

A.

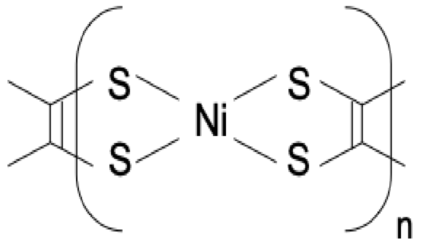

B.

Figure S6. Chemical structures of (A) N2200 and (B) poly(NiETT)
